# Hypertriglyceridemic Waist Phenotype and Changes in the Fasting Glycemia and Blood Pressure in Children and Adolescents Over One-Year Follow-Up Period

**DOI:** 10.5935/abc.20170067

**Published:** 2017-07

**Authors:** Priscila Ribas de Farias Costa, Ana Marlúcia Oliveira Assis, Carla de Magalhães Cunha, Emile Miranda Pereira, Gabriela dos Santos de Jesus, Lais Eloy Machado da Silva, Wilanne Pinheiro de Oliveira Alves

**Affiliations:** 1 Universidade Federal da Bahia (UFBA); Salvador, BA - Brazil; 2 Pós-graduação em Alimentos, Nutrição e Saúde da UFBA; Salvador, BA - Brazil; 3 Universidade Federal do Recôncavo da Bahia, Salvador, BA - Brazil

**Keywords:** Hypertriglyceridemic Waist, Phenotype, Glycemic Index, Fasting, Blood Pressure, Child, Adolescent, Cohort Studies

## Abstract

**Background:**

The hypertriglyceridemic waist (HTW) phenotype is defined as the simultaneous presence of increased waist circumference (WC) and serum triglycerides (TG) levels and it has been associated with cardiometabolic risk in children and adolescents.

**Objective:**

The objective was to evaluate the influence of HTW phenotype in the fasting glycemia and blood pressure in children and adolescents over one-year follow-up period.

**Methods:**

It is a cohort study involving 492 children and adolescents from 7 to 15 years old, both genders, who were submitted to anthropometric, biochemical and clinical evaluation at the baseline, and also after 6 and 12 months of follow-up. Generalized Estimating Equation (GEE) models were calculated to evaluate the longitudinal influence of the HTW phenotype in the glycemia and blood pressure over one-year.

**Results:**

It was observed a prevalence of 10.6% (n = 52) of HTW phenotype in the students. The GEE models identified that students with HTW phenotype had an increase of 3.87 mg/dl in the fasting glycemia mean (CI: 1.68-6.05) and of 3.67mmHg in the systolic blood pressure (SBP) mean (CI: 1.55-6.08) over one-year follow-up, after adjusting for confounding variables.

**Conclusions:**

The results of this study suggest that HTW phenotype is a risk factor for longitudinal changes in glycemia and SBP in children and adolescents over one-year follow-up period.

## Introduction

The hypertriglyceridemic waist phenotype (HTW) is defined as the simultaneous presence of increased waist circumference (WC) and serum triglycerides (TG) levels.^1^ This phenotype has been associated with the cardiometabolic risk, with elevated insulin, apolipoprotein B, C-reactive protein and LDL cholesterol levels, increasing the risk of coronary artery disease.^2^

The prevalence of HTW phenotype has been widely investigated. A meta-analysis performed by Ren et al. (2016)^3^ demonstrated variation in the prevalence of the HTW phenotype from 4% to 47%, with combined prevalence of 19% (95% CI 14-24%). Esmaillzadeh et al.^4^ verified prevalence of 6.5% for HTW in Iranian adolescents (7.3% in boys and 5.6% in girls). In Brazil, transversal studies with adolescents from 10 to 19 years old identified HTW prevalence fluctuating from 6.4% to 20.7%.^5-7^ However, no longitudinal studies involving children and adolescents were identified in the literature.

Evidences suggest that individuals with HTW phenotype are more likely to develop metabolic syndrome and risk factors for cardiovascular diseases^4,8-10^. Among these factors, there are increased glycemia and systemic hypertension, characterized by increased and sustained blood pressure, with multifactorial determination^8^. Therefore, HTW is a simple and reliable indicator to detect these diseases and metabolic risks associated to visceral obesity^9^, and may become a practical, feasible, and low-cost approach, especially in Primary Care.

Given the above, and considering the absence of cohort studies involving the subject, this study aimed to assess the influence of the hypertriglyceridemic waist phenotype on the fasting glycemia and blood pressure of students after one-year follow-up.

## Methods

### Sample and study design

This is a cohort study including 492 children and adolescents from 7 to 15 years old, both male and female, from 10 public, urban and part-time schools. It was conducted a simple random sampling, selecting students of each school from a list of elementary school children registered with the Municipal Education Department of a municipality of Bahia, Brazil, in 2006. This sample has power of 97.6% to detect a 10% change in the mean fasting glycemia of the participants, during a 12 months period, considering the mean of 90.2 mg/dL ± 0.3SD.^7^ For systolic blood pressure (SBP), the sample has power of 95% to detect alteration of 10% in the average values, considering the mean of 111.1 mmHg ± 12.9 SD; and power of 94% to detect a change of 10% in the mean of diastolic blood pressure (DBP), considering an average of 70.3 ± 9.3SD.^11^ Calculations of sample power (1-β) were based on the significance level of 5% and two tailed-tests, indicating that this sample size is sufficient to carry out unbiased estimates of the parameters of the studied population.

All the measurements were collected in the beginning and after 6 and 12 months follow-up.

### Exclusion criteria

State of pregnancy, lactation or physical disabilities that prevented anthropometric evaluation were adopted as exclusion criteria. However, these conditions were not identified among the selected students.

### Anthropometric evaluation

In order to measure the weight, it was used a Filizola® portable weighing machine with capacity for 150kg and 100 g precision, allowing the variation of 100g. Height was measured by a stadiometer brand Leicester Height Measure, with the measure performed in the closest millimeter. Both measures were performed in duplicate and the mean of the two measures was adopted as final measure.^12^ The anthropometric state was assessed by Body Mass Index (BMI) by age, using as reference, the recommendations of World Health Organization^13^ for individuals from 5 to 19 years old.

The waist circumference (WC) was measured at the midpoint between the iliac crest and the outer face of the last rib. The measures were performed in duplicate and the average of these two measures was adopted as definitive. The cut off adopted to classify abdominal fat excess was the 90th percentile of the own sample, as proposed by Freedman et al. (1999).^14^

### Sociodemographic and lifestyle data

The person responsible for the child/adolescent referred this information. The demographic variables included the student gender and age. The socioeconomic status included number of rooms in the house and people who lived there, the main source of illumination and the occupation of the head of the family. These variables originated the socioeconomic index. The status of household water supply, the source of drinking water and the destination of the garbage and waste were used to compose the environmental index. These variables had responses varying from 0 (worst condition) to 4 (best classification). Thus, the socioeconomic and environmental indexes were ranging from 0 to 16 and were categorized into terciles. Despite knowing that the maternal education is associated to socioeconomic conditions, this variable was not included in the socioeconomic index and it was assessed individually, once this also represents the cultural and dietary aspects of the society where the individual is inserted.

The physical activity level was assessed by a questionnaire structured with questions referring to the frequency of physical activities that were not included in the pedagogical content of the school, which is performed once a week. Therefore, the practice of two or more days of physical activity outside the school classifies the student as active; and less than two days of physical activity outside classifies the student as less active / sedentary.

### Blood pressure

For blood pressure (BP) measurement it was adopted the technique recommended by the 4^th^ Brazilian Guidelines on Hypertension. The BP value was classified according to the *National High Blood Pressure Education Program Working Group on High Blood Pressure in Children* (2004),^15^ considering high pressure levels for children and adolescents BP > p90, according to age, gender and height percentile.

### Biochemical exams

The blood collection was performed in the morning observing at least 12-hours fasting, 10 mL of blood were collected by venepuncture and deposited in sterile and disposable vacutainer tubes (BD^®^), without anticoagulant. The blood was centrifuged at 3000 rpm for 5 minutes for posterior serum separation, which was used for biochemical determinations. The biochemical parameters were performed at the Reference City Laboratory, provided by the Health Department. The triglyceride and glycemia values were determined by the enzymatic method. For the glycemia classification, the recommendation of the Brazilian Diabetes Society (2009)^16^ was adopted, which characterizes the adequate conditions (≤ 100mg/dL) and increased conditions (>100 mg/dL). Triglyceride values < 100 mg/dL were considered adequate.^17^

### Hypertriglyceridemic waist phenotype

The HTW phenotype was defined by the presence of increased waist circumference (> 90 percentile by age and sex of the sample itself) and increased serum triglycerides (> 100 mg/dL), simultaneously.^4^

### Statistical Analysis

Prevalence of categorical data and measure of mean and standard deviation for continuous variables were calculated. The comparison of outcome variables and other variables of interest according to exposition variable was performed using Pearson^2^- square test. The p-value lower than 0.05 was adopted as significant level.

The mean and standard deviation of the glycemia, the systolic and the diastolic BP were calculated. The Shapiro-Wilk normality test was applied, and the statistic indicates normality^1^ when close to one.^8^ Distributions of the mean values of glycemia, the systolic and the diastolic BP according to HTW phenotype at baseline and after the follow-up were calculated using the statistical test of mean comparison for continuous variables (Student t test for equal or unequal variances), adopting the p-value lower than 0.05 as significant level.^18^

Aiming to assess the influence of the HTW phenotype on fasting glycemia and BP of the students during 12 months, models of Generalized Estimating Equations (GEE) were built, which is adequate for continuous answers and repeated measures, reflecting the relationship between the variable and independent responses, considering the correlation between the measures in each moment of time. Furthermore, GEE model does not require normality assumption. For the present study, the correlation matrix chosen was the autoregressive, considering that the measures have an autoregressive relationship in function of time.^19^

Initially, the univariate analysis was conducted, aiming to select the variables candidate to the multivariate model, selecting those with p value lower than 20%. These variables were included in the model as covariables. In the final model, the remaining variables were those that presented significance level lower than 5%.

To assess the data adjust of the model, the criterion of quasi-likelihood under the corrected independence model (QIC_c_) was used, which is a modification of the *Akaike's information criterion* (AIC) for GEE analysis. The QIC is calculated from the comparison of the quasi-likelihood of the independence model with that of the complete model. The lower the QIC, the better the model adjustment.^20^

All the statistical analyses were performed in the statistical package *Stata/IC for Mac* (*StataCorp, College Station*), version 12.0.

### Ethical Aspects

The protocol of the present study was approved by the Nutrition Ethics Committee of the School of Nutrition by the number 03/06.

The participation of the student in the study depended on a written authorization of the parents and/or responsible person. After receiving the letter-invitation, knowing the study objectives and agreeing with the insertion of the minor in the investigation, the parents and/or responsible person signed the informed consent form (ICF).

## Results

In Table 1 are presented the sociodemographic, clinical and anthropometrical characteristics of the students, according to the HTW phenotype. There was higher prevalence of the HTW phenotype among students with lower socioeconomic status (14,3%), with fasting glycemia above 100 mg/dL (22,9%) and with excess weight, according to the BMI (31,1%).

**Table 1:** Sociodemographic, anthropometric and clinical characteristics at baseline of students from a city of Bahia, Brazil, 2006

	HTW Phenotype N (%)			
	Total N	Absent	Present	p value
**Gender**				
– Female	492	255 (88.8)	32 (11.1)	0.620
– Male		185 (90.2)	20 (9.8)	
**Age**				
– <10 years	492	124 (90.5)	13 (9.5)	0.620
– ≥ 10 years		316 (89.0)	39 (11.0)	
**Environmental index**				
– 3rd tercile	492	177 (88.9)	22 (11.1)	0.770
– 1st e 2nd terciles		263 (89.8)	30 (10.2)	
**Socioeconomic index**				
– 3º Tercile	492	206 (94.1)	13 (5.9)	0.003*
– 1º e 2º Terciles		234 (85.7)	39 (14.3)	
**Maternal education**				
– ≥ 6 years	439	171 (89.5)	20 (10.5)	0.090
– < 6 years		233 (93.9)	15 (6.1)	
**Physical activity**				
– Active	492	133 (86.9)	20 (13.1)	0.113
– Low active/sedentary		284 (91.6)	26 (8.4)	
**Blood pressure**				
– < P90	492	324 (91.0)	32 (9.0)	0.467
– ≥ P90		117 (86.0)	19 (14.0)	
**Glycemia**				
– <100mg/dL	492	413 (90.4)	44 (9.6)	0.01*
– ≥ 100 mg/dL		27 (77.1)	8 (22.9)	
**BMI/age**				
– < P85	492	369 (94.8)	20 (5.2)	0.000*
– ≥ P85		71 (68.9)	32 (31.1)	

* Significant p value for Pearson chi-square; HTW: hypertriglyceridemic waist.

Considering the initial sample of this study, the loss of 37 (7.5%) individuals was registered after one year of follow-up. The analysis of the sociodemographic and clinical data indicated that there were no significant statistical differences among these factors for the students lost and those that continued in the study (data not shown).

The outcome variables (fasting glycemia, systolic BP and diastolic BP) presented normal distribution, according to the Shapiro-Wilk test; being applied at the mean comparison t-test. Thus, the Table 2 shows the mean values of the variables of interest at the beginning and at the end of the follow-up, according to the HTW phenotype. It was observed that in the beginning of the study, the students with HTW phenotype presented higher mean values of glycemia and systolic BP (p = 0,003 and p = 0,03, respectively). After one year of follow-up, it was identified that individuals with HTW phenotype had higher mean values of glycemia than those without this phenotype (p = 0,04).

**Table 2:** Mean comparison test for the variables of interesting according to the hypertriglyceridemic waist phenotype at the baseline after one-year follow-up in students from a city of Bahia, Brazil, 2006

	Baseline			After one-year follow-up		
	HTW(-)	HTW(+)	p value	HTW (-)	HTW (+)	p value
	Mean (SD)	Mean (SD)		Mean (SD) final	Mean (SD) final	
Glycemia (mg/dL)	81.8 (10.2)	86.0 (11.7)	0.003	83.5 (10.3)	86.1 (11.6)	0.04
Systolic BP (mmHg)	101.3 (12.0)	105.1 (12.1)	0.03	101.3 (11.8)	104.1 (11.0)	0.10
Diastolic BP (mmHg)	64.3 (10.1)	66.9 (10.3)	0.09	64.5 (10.6)	66.1 (10.6)	0.09

HTW: hypertriglyceridemic waist.

The GEE models, constructed to evaluate the influence of HTW phenotype in the glycemia and blood pressure of the students after one-year follow-up, are presented in Table 3. For fasting glycemia, it was observed that students with the presence of HTW phenotype had the increase of 3.11 mg/dl in the mean of fasting glycemia after one-year follow-up, when compared to individuals without the HTW phenotype. Adjusting by gender, age, maternal education, socioeconomic status, BMI and physical activity level, the increase in the mean of fasting glycemia after one-year follow-up was 3.87 mg/dl (p = 0,001).

**Table 3:** Models of Generalized Estimating Equation for the relationship between HTW phenotype and fasting glycemia, systolic and diastolic blood pressure after one-year follow-up in students from a city of Bahia, Brazil, 2006

- HTW phenotype Absent PresentQIC_c_^***^	**Fasting glycemia** Coefficient (95% CI); p value^*^	
	**Crude** Reference 3.11 (1.35-4.86); 0.001 128.540	**Final model**^†^ Reference 3.87 (1.68-6.05); 0.001 112.716
- HTW phenotype Absent PresentQIC_c_^‡^	**Systolic Blood Pressure** Coefficient (95% CI); p value^*^	
	**Crude** Reference 2.97 (-0.11- 6.06); 0.06 125.375	**Final model**^†^ Reference 3.67 (1.55-6.08); 0.02 118.265
- HTW phenotype Absent PresentQIC_c_^‡^	**Diastolic Blood Pressure** Coefficient (95% CI); p value^*^	
	**Crude** Reference 1.43 (-1.20- 4.05); 0.28 90.724	**Final model**^†^ Reference 1.45 (-1.20- 4.10); 0.29 83.872

Sample size- 492; HTW: hypertriglyceridemic waist.

* Generalized Estimating Equation – GEE;

† Adjusted by gender, age, maternal education; socioeconomic status and level of physical activity;

‡ QICC: quasi-likelihood corrected under the criterion of Independence model for.

For the SBP, students with HTW phenotype had increase of 2.97 mmHg in the average of this measure after one-year follow-up, when compared to individuals without this phenotype. This raise in the SBP mean increased for 3.67 mmHg in individuals with HTW phenotype after one-year follow-up when adjusting by sociodemographic variables, BMI and physical activity level (p = 0,02).

The presented models were well adjusted to the data, according to the QICc criterion, considering that there was a reduction of this indicator in the final models when compared to the crude models (Table 3).

Significant statistical changes were not identified in the DBP mean in children and adolescents with HTW phenotype after the 12-months follow-up.

## Discussion

The results of this investigation indicate higher prevalence of HTW phenotype among students with lower socioeconomic status, with altered fasting glycemia and weight excess, indicating important environmental component in this phenomenon. Furthermore, the presence of HTW phenotype favored the increase of mean values of glycemia and systolic BP after one year, especially after the adjustment by sociodemographic variables, BMI and physical activity level.

The prevalence of HTW phenotype identified in this study was higher than that found in children and adolescents in Iran (3.3% and 8.5%, respectively)^4,21^ and in the United Kingdom (varying from 6.3% to 8.2%).^22^ Investigations involving Brazilian adolescents found the occurrence of HTW phenotype from 2.6% to 20,7%.^5,6^ These differences among prevalence of HTW phenotype can reflect the lack of world standardization for measuring the waist circumference, and the serum concentrations of triglycerides for the age, moreover the variations of the cutoff used to classify the HTW phenotype, which is an obstacle to the comparison of the investigations. Besides, the differences of lifestyle, genetic background and ethnicity interfere with the accumulation of abdominal fat and can explain the divergent results of the HTW phenotype prevalence.^21,23,24^

Data from this study indicate that the HTW phenotype increases the fasting glycemia mean after one-year follow-up when compared to the individuals without the HTW phenotype. This is an important clinical situation among the studied population, because the glycemia is related to visceral obesity, favoring the higher risk of developing others chronic and non-communicable diseases with expression in adult life.^25,26^ However, different studies did not find the relationship between HTW phenotype and fasting glycemia in children and adolescents^4,7,21^ but it worth noting the transversal design of these investigations.

The increase of HTW phenotype is associated with metabolic modifications. It is suggested that with increased values of the waist circumference, there are greater concentrations of free fatty acids, especially in the liver, muscle and pancreas, resulting from lipolysis of triglycerides. The excess of free fatty acids provides negative feedback of the glycogen synthase, which can induce the peripheral resistance to insulin and glucose intolerance, in both muscle and liver^27,28^ which can explain the longitudinal relationship between HTW phenotype and glycemia identified in this study.

Even though some evidences identified the association between HTW phenotype and lipid alterations and in the hyperglycemia in children and adolescents, the relationship of the HTW phenotype and blood pressure levels is still not established for this age, because of the reduced number of publications that test this association and the limitations of the transversal study designs. In the study performed by Kelishadi et al.^23^ SBP greater than 90th percentile for the age, gender and height was observed in 8.8% of the boys and in 17.6% of the girls with HTW phenotype, while diastolic blood pressure was 4.4% and 9.6%, respectively. Bailey et al.^22^ observed higher means of DBP in children and adolescents with HTW phenotype.

The prevalence of high BP in people with HTW phenotype is 2 to 3 times greater when compared to those who do not present this phenotype.^4,20^ This association is usually weakened or becomes statistically insignificant when BMI adjusts are conducted, suggesting that obesity can influence intensely the blood pressure and the distribution of isolate fat.

Although it is a cohort study, it is known that it is not possible to completely establish a causal relationship, being necessary to undertake confirmatory studies about these relationships identified in the present study. Moreover, maybe the follow-up period was not enough to identify the outcome variables in this population. However, the study was methodologically well designed, robust statistical techniques were adopted, and, in addition, the results are biologically plausible and consistent with the scientific evidence on this theme.

Therefore, in the present study, the longitudinal relationship between HTW phenotype and SBP was stronger after one-year follow-up, even when adjusted by BMI. This can be the reflection of the strength of a cohort study and it suggests that the presence of the phenotype is characterized as risk factor for progressive increase of SBP in this group. This relationship can be related to the presence of higher serum level of insulin in people with abdominal obesity, regardlessly the body weight,^29^ since the insulin hormone induces various signals that promote increase of BP, which includes induction of vasoconstriction and proliferation of smooth muscle cells in blood vessels; promotion of pro-inflammatory activity; stimulus of renal absorption of sodium and sympathetic response.^30-32^

## Conclusions

The results of this study suggest that HTW phenotype is a risk factor for longitudinal changes in glycemia and SBP in children and adolescents. Considering that the components of this phenotype are already present early in life, the monitoring of HTW phenotype should be adopted in the pediatric group, since it is a simple and low-cost screening tool to identify cardiometabolic risk, and the diagnosis of the risk provided prematurely can favor the nutritional and lifestyle interventions, promoting health and preventing chronic non-communicable diseases in the later age.
